# An Exploratory Study of a Dimensional Assessment of the Diagnostic Criteria for Autism

**DOI:** 10.1007/s10803-020-04474-8

**Published:** 2020-03-26

**Authors:** Mark Brosnan

**Affiliations:** Centre for Applied Autism Research, Department of Psychology, University of Bath, Bath, BA2 7AY UK

**Keywords:** Autism assessment, Diagnostic criteria, Autistic-like traits

## Abstract

Prevalence rates of autism based upon child samples have shown a consistent increase over the past three decades, suggesting that many autistic adults are undiagnosed. Adult diagnostic pathways typically are initiated with measures of autistic-like traits. Whilst autistic-like traits represent a continuous dimension across the general population, autism is a categorical diagnosis and the relationship between the two is unclear. A self-report dimensional reflection upon the two diagnostic criteria for autism was developed and reflected upon by 1076 participants embedded within two online surveys. Those with an informal (self) diagnosis of autism self-reported comparable social difficulties but fewer restricted and repetitive behaviour difficulties than those with a formal diagnosis of autism. The new items also significantly correlated with autistic-like traits.

## Introduction

Autism Spectrum Disorder (hereafter autism) is a neurodevelopmental condition defined by persistent difficulties across multiple contexts within two distinct domains: (1) social communication and interaction and (2) restricted and repetitive patterns of behaviours, activities or interests (APA 2013; WHO 2018). Studies in Asia, Europe, and North America have identified an average prevalence of autism of between 1 and 2% (Baio et al. 2018). In the USA, substantial increases in prevalence have been identified over the past three decades, which have been attributed to greater autism awareness and changing diagnostic criteria, which have reduced the number of missed cases (see Baio et al. 2018; Christensen et al. 2018). As these prevalence estimates are based upon child populations, this would suggest that there are a number of adults on the autism spectrum who are undiagnosed, which is compounded by a lack of adult-focused autism research (see Camm-Crosbie et al. 2019; Cashin et al. 2016; Pellicano et al. 2014; Milton and Bracher 2013; Warner et al. 2019).

Whilst a diagnosis of autism is categorical, either a diagnosis is present or it is not, people have reported an autism social identity, some of whom do not have a formal diagnosis of autism (Cooper et al. 2017). Cooper et al. report that around one fifth of their sample identifying as autistic did not have a formal diagnosis from a professional, rather they perceived themselves to be ‘autistic-like’. Autistic-like traits refer to behavioural traits such as social imperviousness, directness in conversation, lack of imagination, affinity for solitude, and difficulty displaying emotions (Gernsbacher et al. 2017). Autistic-like traits are argued to vary continuously across the general population, with studies reporting that groups with a formal diagnosis of autism typically have higher levels of autistic-like traits than non-autistic comparison groups (see Ruzich et al. 2015 for meta-analysis). Autistic-like traits are typically assessed through self-report measures such as the 50-item Autism Spectrum Quotient (AQ: Baron-Cohen et al. 2001; see also Baghdadli et al. 2017). Ruzich et al.’s (2015) meta-analysis of responses to the AQ from almost 7000 non-autistic and 2000 autistic respondents identified that non-autistic males had significantly higher levels of autistic-like traits than non-autistic females, and that autistic people had significantly higher levels of autistic-like traits compared to the non-autistic males (with no sex difference within the autistic sample).

In the UK, the National Institute of Health and Clinical Excellence (NICE) guidelines recommend using a 10-item version of the AQ (Allison et al. 2012; Pilling et al. 2012; Wilson et al. 2014) as a screen for adults (without intellectual disability) who may have autism, which has a clinical cut-off of 6 (out of 10). The NICE guidelines propose that if an adult scores above cut-off on the AQ10, offer a comprehensive assessment for autism.^1^ There are items on the AQ10 that do not refer directly to diagnostic criteria, rather to cognitive theoretical accounts of autism such as Weak Central Coherence Theory (‘I usually concentrate more on the whole picture, rather than the small details’ [reverse scored]), Theory of Mind (‘When I’m reading a story I find it difficult to work out the characters’ intentions’) or Executive Functioning Theory (‘I find it easy to do more than one thing at once’ [reverse scored]). The aetiology of autism and high levels of autistic-like traits is therefore found to be similar, with more similarities than differences (Bralten et al. 2018; Lundström et al. 2012; Ronald and Hoekstra 2011; see also Massrali et al. 2019).

Importantly, however, there are also differences between those with and without a formal diagnosis of autism who scored above the cut-off on the AQ (Ashwood et al. 2016; Lundqvist and Lindner 2017; Payne et al. 2019) and autistic-like traits (measured by the AQ) have been found to not consistently be predictive of formal autism diagnosis (Bishop and Seltzer 2012; Conner et al. 2019; Sizoo et al. 2015). Frith (2014) speculates that heightened awareness of autism, combined with stretching the diagnostic boundaries, may have resulted in individuals with problems in social relationships and other features that are reminiscent of autism thinking they are autistic, when this may be part of neurotypical individual variation. Frith asks: ‘Should we believe only in continua and quantitative differences, or by contrast, in categorical and qualitative differences?’ (p. 745).

Abu-Akel et al. (2019) recently proposed the co-existence of a mixed categorical and dimensional architecture within the autism spectrum, suggesting that dimensional and categorical classifications of autism need not be mutually exclusive and that higher autistic-like traits may reflect greater genetic liability for a formal diagnosis of autism. If dimensional and categorical classifications of autism are complementary, it should be possible to develop a dimensional assessment that directly reflects the diagnostic criteria for autism. The AQ scale (50 and 10 item) is made up of five subscales, namely social skills, communication, attention to detail, attention switching and imagination which, as noted above, are not all synonymous with the diagnostic criteria for autism. Developing a measure that directly relates to the diagnostic criteria for autism may ultimately lead to a more reliable and valid initiation of autism diagnostic pathways. This exploratory study therefore sought to develop a dimensional assessment of the diagnostic criteria for autism. The aim of the study was to explore the self-reporting of items that directly reflect the diagnostic criteria for autism on a dimensional assessment and compare them to existing dimensional assessments of autistic-like traits.

## Method

### Design

Participants were asked their sex (male/female/non-binary), age and diagnostic status (Do you have a formal clinical diagnosis of autism (i.e. from a qualified health professional); an informal diagnosis of autism (from a friend or colleague or self-diagnosis, but do NOT have a formal clinical diagnosis from a qualified health professional); none of the above. These groups are referred to as autism-diagnosed, autism-undiagnosed and no-autism, respectively. The autism-undiagnosed category has been used for online surveys of the autistic community to capture those who do not have a formal diagnosis (e.g.Au-Yeung et al. 2019; Kapp et al. 2013). Wording was developed for each of the two diagnostic criteria (social and RRB items), with examples that related to each of the descriptors,^2^ which enabled responding upon a continuous scale (see Appendix 1).

### Procedure

These two questions were responded to within two online courses, one that targeted autistic people, and one that targeted the general population to obtain larger numbers of both those with and without autism. Survey 1 was embedded in an online survey exploring alcohol consumption by autistic people (reported elsewhere). Survey 2 was embedded in an online course on autism (FutureLearn.com) and was identical to Survey 1 with the addition of the AQ10 (Allison et al. 2012) after the social and RRB items. From the literature above, it was anticipated that the general population would provide a range of AQ scores that could be correlated with the two questions developed for this study (Survey 2), whereas AQ scores may have reduced variability within an autistic population (Survey 1). The AQ10 has a reported Cronbach's alpha 0.85. A clinical cut-off of 6 or more is proposed, which has sensitivity of 0.88, specificity of 0.91, and a positive predictive value of 0.85 (Allison et al. 2012). The self-assessment was presented as an option for those who would like to reflect upon the diagnostic criteria for autism and how they applied to themselves. The self -assessment was very early in both courses. This was not mandatory and those taking the survey/course could continue without answering these items. Participants did not have to pay to take the course and were not compensated for responding to the two self-assessment items.

Ethical approval was granted by the Psychology Reserach Ethics Committe, University of Bath. All responses were confidential and anonymous and participants could withdraw at any point, returning to the online survey/course. The online survey/course provided advice and support concerning autism and signposted further support should it be required.

### Participants

715 participants took online Survey 1. 97 did not complete the survey, one was removed for reporting an age of under 16 and one participant did not consent for their data to be used, leaving 616 (86%). There were 148 male, 400 female and 68 non-binary participants with a mean age of 38.71 (sd = 13.06, range 16–89). 424 were autism-diagnosed, 128 autism-undiagnosed, and 63 no-autism. 543 participants responded to Survey 2, of which 20 did not provide consent for their data to be analysed, four were removed for being under 16 and 59 did not complete any questions. This left 460 (85%) participants. There were 55 males, 398 females, four non-binary (and three missing sex data) participants with a mean age of 44.20 (sd = 13.74, range 16–75). There were 16 autism-diagnosed, 82 autism-undiagnosed and 362 no-autism. Overall there were 1076 participants, comprising 798 females (74.2%), 203 males (18.9%) and 72 non-binary (6.7%, with three missing) with a mean age of 41.0 years (sd = 13.64; range = 16–89, 14 missing). 440 identified as autism-diagnosed (40.9%), 210 as autism-undiagnosed (19.5%) and 425 as no-autism (39.5%; 1 missing).

## Results

The means of both items are reported in Table 1 (both having a median of 2) and the two items correlated with each other (r(1019) = 0.70, p < 0.001). A MANOVA was run in which the dependent variables were the social and RRB items, sex and diagnostic status were the independent variables. Survey (1 or 2) was added as a dummy variable. There were no significant differences by sex on the social item (F(2,1015) = 2.63, p = 0.072) nor the RRB item (F(2,1015) = 0.20, ns), see Table 1. Diagnostic status had a significant impact upon both social (F(2,1015) = 9.16, p < 0.001) and RRB (F(2,1015) = 18.19, p < 0.001) items, see Table 1. Tukey HSD post hoc tests revealed that for the social item, the no-autism group significantly differed from the autism-diagnosed and the autism-undiagnosed groups (both p < 0.001). The autism-diagnosed and autism-undiagnosed groups did not significantly differ from each other (p = 0.062). For the RRB item, all three groups significantly differed from each other: The no-autism group significantly differed from the autism-diagnosed and the autism-undiagnosed groups (both p < 0.001) and the autism-diagnosed and autism-undiagnosed groups also significantly differed from each other (p = 0.024). There were no significant interactions between sex and diagnostic status (both p > 0.05). There was also not a significant main effect for survey, nor significant two-way interactions with survey, nor a significant three-way interaction with survey and sex and diagnostic status (all p > 0.05). As an additional check, the MANOVA analysis above was rerun for each survey separately and the pattern of results was the same, except that there was a significant sex difference for the social item for Survey 1 (F(2,604), p = 0.018) but not Survey 2 (F(2,394) = 0.84, ns) and the sex by diagnostic status interaction was also significant for the social item (F(4,604) = 2.74, p = 0.028). For the no-autism group in Survey 1, males scored higher (mean = 2.25, sd = 1.34) than females (1.43. sd = 1.17).

**Table 1:** Means (and standard deviation) for the Social and RRB items by sex and diagnostic status

	Social item	RRB item
Overall	2.31 (1.19)	2.16 (1.30)
Male	2.63 (1.10)	2.39 (1.27)
Female	2.16 (1.20)	2.03 (1.31)
Non-binary	3.03 (0.84)	2.86 (0.95)
Autism-diagnosed	2.95 (0.82)	2.91 (0.88)*
Autism-undiagnosed	2.77 (0.84)	2.69 (0.95)*
No-autism	1.36 (1.07)*	1.03 (1.05)*
Below cut-off	1.29 (1.01)*	0.96 (1.00)*
Above cut-off	2.87 (0.81)*	2.61 (0.99)*

*Significantly different from other categories in column

**Fig. 1:**
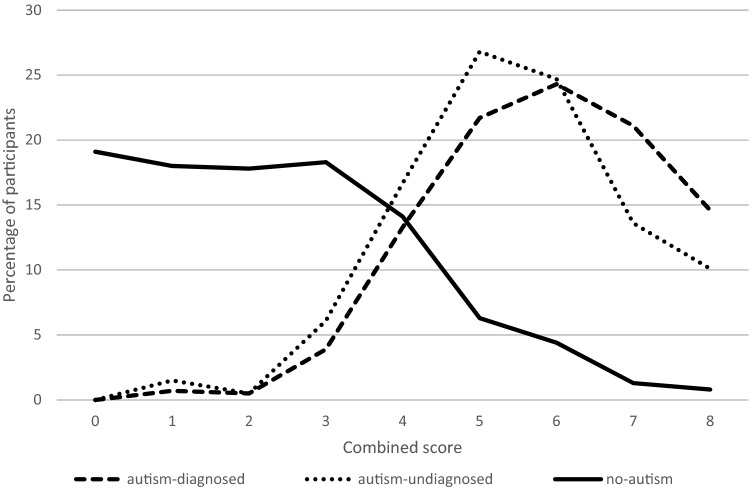
Proportion of participants obtaining each combined score by diagnostic status

For Survey 2, the AQ10 correlated positively with the social item (r(405) = 0.66, p < 0.001) and the RRB item (r(405) = 0.68, p < 0.001). The AQ10 correlated with the social item for the subgroups as follows: autism-diagnosed (r(15) = 0.51, p = 0.05); autism-undiagnosed (r(70) = 0.46, p < 0.001); no-autism (r(320) = 0.47, p < 0.001) and for the RRB item as follows: autism-diagnosed (r(15) = 0.19, p = ns); autism-undiagnosed (r(70) = 0.52, p < 0.001); no-autism (r(320) = 0.46, p < 0.001). Taking the AQ10 cut off of 6, 13 (81%) of those who were autism-diagnosed were above cut off, 59 (72%) of those autism-undiagnosed were above cut off and 19 (5%) of those with no-autism were above cut off. In addition, both the social and the RRB items significantly positively correlated with every AQ10 item separately (all p < 0.01; *r*s mostly 0.4–0.6). Table 1 highlights the means on the two items for those above and below cut-off which were significantly different from each other (social item t = 15.13, p < 0.001; RRB item t = 13.59, p < 0.001). The sum of the two items are combined in Table 2, which highlights that around 80% of the no-autism group have a score between 0 and 3 and around 80% of the autism groups have a score between 5 and 8, with both groups having around 15% with a score of 4, see Fig. 1. The combined scale had a Chronbach’s Alpha of 0.82.

**Table 2:** Number (and percentage) of each combined score by diagnostic status

	Autism-diagnosed (%)	Autism-undiagnosed (%)	No-autism (%)
0	0	0	73 (19.1)
1	3 (0.7)	3 (1.5)	69 (18.0)
2	2 (0.5)	1 (0.5)	68 (17.8)
3	17 (3.9)	12 (6.1)	70 (18.3)
4	58 (13.3)	33 (16.7)	54 (14.1)
5	95 (21.7)	53 (26.8)	24 (6.3)
6	106 (24.3)	49 (24.7)	17 (4.4)
7	92 (21.1)	27 (13.6)	5 (1.3)
8	64 (14.6)	20 (10.1)	3 (0.8)

## Discussion

This is the first study to explore how a dimensional self-reflection upon the diagnostic criteria for autism related to dimension assessments of autistic-like traits in adults. Firstly, participants were able to respond to the questions, and overall there were no significant sex differences and no difference due to which survey participants were drawn from. Secondly, those with a formal diagnosis of autism did not significantly differ from those with an informal (self) diagnosis of autism on the social item but did score significantly higher on the RRB item. Both groups scored significantly higher than the no-autism group on both items. Thirdly, these self-reflections upon the diagnostic criteria were in accordance with the AQ10 scale assessment of autistic-like traits.

Further analysis did highlight that there was some evidence of a sex difference favouring females in the no-autism group for the social item that did not extend to the other groups (in Survey 1). This male–female sex difference in a non-clinical population not extending to autistic populations is consistent with the literature (e.g. Ruzich et al. 2015). Sex differences were not identified for the RRB item, however. Given the highest scores were reported for those identifying as non-binary (see Table 1), it is important for future research to consider the options available for respondents to report their sex and gender (e.g. see Cooper et al. 2018). Only 2 of the 72 (3%) identifying as non-binary were from the no-autism group. The non-binary group was retained in the analysis as it made up 6.7% of the sample. Other authors have removed this group as it can be small (e.g. Kapp et al. 2013) but rerunning the present study’s analysis without the non-binary group does not change the pattern of results.

A fifth of the present study self-identified as autistic, which is a similar proportion to other research (Cooper et al. 2017), and may reflect older people having missed formal diagnoses (current prevalence rates being based on child samples, Baio et al. 2018). Consistent with this, both formally and informally diagnosed groups did not significantly differ from each other on the social item. However, inconsistent with this, these two groups did significantly differ from each other on the RRB item. It may be, therefore that the RRB aspect of the diagnostic criteria differentiates those who meet diagnostic criteria, and this aspect of the diagnostic criteria should be a feature of autism awareness for those considering embarking on an autism diagnostic pathway. Importantly, however, the difference in the RRB item was relatively small (p = 0.024) and the social item had a trend in the same direction, albeit non-significant (p = 0.062). When combining the scores on the two items, the vast majority of those identifying as autistic (with or without a diagnosis) experienced the behaviours associated with the diagnostic criteria in most or all situations and those self-reporting no-autism experienced them rarely or never. Around 15% of both those identifying as autistic and those not identifying as autistic, reported experiencing behaviours associated with diagnostic criteria in some situations. It is interesting that both groups (with and without autism) can report similar levels of behaviours related to the diagnostic criteria and may be due to differing levels of insight between autistic and non-autistic groups.

It has been found, however, that autistic people have the insight to accurately self-report data relating to personality traits and mental health which are comparable with non-autistic groups (Ozsivadjian et al. 2014; Schriber et al. 2014). Whilst parent-report and self-report of autistic-like traits have been found to vary in absolute terms (by around 7%), they still highly correlate with each other (Baron-Cohen et al. 2001; Johnson et al. 2009). These studies have separately found that when differences appear, self-report can be both higher or lower than parent-report. Bishop and Seltzer (2012) propose that insight for self-report may be affected by level of cognitive functioning, as those with higher IQ may be more aware of their difficulties. A limitation of this study is that measures of IQ were not taken. A relatively high level of literacy was required to access the survey and it is likely that participants had IQs within the normal range or above. The AQ10 screen is intended for adults with suspected autism who do not have a learning disability, and it is important for future research to consider how best to support accurate self-report in those on the autism spectrum with co-occurring intellectual disability. Minimising the number of items and focussing the items directly on the criteria being assessed, as in the present study, may prove a useful starting point for this.

Consistent with Abu-Akel et al. (2019) the significant correlations between both items and each item on the AQ10 suggests that the dimensional assessment of diagnostic criteria relates to the autistic-like traits assessed by the AQ10. Comparisons were made with the AQ10 as this is recommended as a screen for adults (without intellectual disability) by the UK’s NICE guidelines. Future research can explore links with the full AQ (50 items) as well as other measures of autistic-like traits (see Baghdadli et al. 2017, for review). Identifying how such a brief measure relates to assessments such as the ADOS (Lord et al. 2000) is to be welcomed. Within the ADOS assessment, social difficulties are often indexed by a lack of typical behaviours (such as eye contact), whereas RRBs are often indexed by the presence of abnormal behaviours (such as hand flapping), which may make RRBs more difficult to assess within the brief assessment context (e.g., hand flapping may only occur when very excited: Hus et al. 2014). As noted above, explicitly highlighting RRBs as diagnostic criteria may prove useful for individuals considering autism diagnosis and for professionals to explore this aspect of the diagnostic criteria beyond the brief assessment context. This may prove pertinent as the RRB item differed between the autism-diagnosed and-undiagnosed groups and did not differ by sex.

Despite the large numbers in the present study, there are many limitations to this exploratory brief report. The items were self-report and clearly the findings are dependent upon the nature of the samples who opted to take part in the surveys. The samples were taken from those who already had an interest in autism (or alcohol consumption for Survey 1), and this may not be reflective of the population as a whole. As noted above, participation required a relatively high level of literacy and comprehension as well as access to the Internet which may limit the generalisability of the findings. It may be that a participant responded to more than one study, and the autistic sample’s self-report of diagnostic status was not verifiable. Survey 1 comprised more autistic respondents than Survey 2 which explored correlations with the AQ10. The sample was largely female, which was not intended and has been reported by other autism-related online studies (e.g. Au-Yeung et al. 2019; Cassidy et al. 2018; Frazier et al. 2018; Kapp et al. 2013; Richards et al. 2019) and needs to be borne in mind when interpreting the results. Along with the present study, these studies usually report a response rate of around 80% being female for both autistic and non-autistic samples. Frazier et al. attempted to reach as broadly as possible to get a diverse population of stakeholders from the autism community for their study, and report 79.1% of 6004 respondents were female. It is interesting to note for future research that whilst adult-oriented online autism studies can be female dominated, child-oriented studies are usually male dominated.

By definition, two questions will be very general, and the questions are not intended to be diagnostic but may have value for autism awareness in highlighting the diagnostic criteria in an accessible manner. It may be that highlighting the restrictive and repetitive behaviour, interests and activities aspect of the diagnostic criteria for autism will be particularly informative for people with problems in social relationships that are reminiscent of autism but may be part of neurotypical individual variation (after Frith 2014).

## Appendix 1

Please answer the following two questions as honestly and accurately as you can. For each question, please choose the one option that is closest to being true for you in everyday life.

1. How often do you have difficulties with social communication and social interaction with other people? (For example, difficulties with normal back-and-forth social conversation, or making normal eye contact or making friends).[Social item]
2. How often do you have difficulties with restricted and repetitive patterns of behaviours, activities or interests? (For example, difficulties with repetitive movements, or insisting on sameness (or routines), or fixated and intense interests, or very high (or very low) sensitivity to the environment—such as light, sound or texture).[RRB item]

0: Almost never/in almost no situations; 1: Rarely/in rare situations; 2: Sometimes/in some situations; 3: Mostly/in most situations; 4: Almost always/in almost all situations. Scores could range from 0–4 for each item.

## Footnotes

1 https://www.nice.org.uk/guidance/CG142/chapter/1-Guidance#identification-and-assessment.

2 https://www.cdc.gov/ncbddd/autism/hcp-dsm.html.

## References

[CR1] Abu-Akel A, Allison C, Baron-Cohen S, Heinke D (2019). The distribution of autistic traits across the autism spectrum: Evidence for discontinuous dimensional subpopulations underlying the autism continuum. Molecular Autism.

[CR2] Allison C, Auyeung B, Baron-Cohen S (2012). Toward brief “red flags” for autism screening: The short autism spectrum quotient and the short quantitative checklist in 1,000 cases and 3,000 controls. Journal of the American Academy of Child & Adolescent Psychiatry.

[CR3] American Psychiatric Association (2013). Diagnostic and statistical manual of mental disorders.

[CR4] Ashwood KL, Gillan N, Horder J, Hayward H, Woodhouse E, McEwen FS, Cadman T (2016). Predicting the diagnosis of autism in adults using the Autism-Spectrum Quotient (AQ) questionnaire. Psychological Medicine.

[CR5] Au-Yeung SK, Bradley L, Robertson AE, Shaw R, Baron-Cohen S, Cassidy S (2019). Experience of mental health diagnosis and perceived misdiagnosis in autistic, possibly autistic and non-autistic adults. Autism.

[CR6] Baghdadli A, Russet F, Mottron L (2017). Measurement properties of screening and diagnostic tools for autism spectrum adults of mean normal intelligence: A systematic review. European Psychiatry.

[CR7] Baio J, Wiggins L, Christensen DL, Maenner MJ, Daniels J, Warren Z, Durkin MS (2018). Prevalence of autism spectrum disorder among children aged 8 years—autism and developmental disabilities monitoring network, 11 sites, United States, 2014. MMWR Surveillance Summaries.

[CR8] Baron-Cohen S, Wheelwright S, Skinner R, Martin J, Clubley E (2001). The Autism-Spectrum Quotients (AQ): Evidence from Asperger Syndrome/High-Functioning Autism, males and females, scientists and mathematicians. Journal of Autism and Developmental Disorders.

[CR9] Bishop SL, Seltzer MM (2012). Self-reported autism symptoms in adults with autism spectrum disorders. Journal of Autism and Developmental Disorders.

[CR10] Bralten J, Van Hulzen KJ, Martens MB, Galesloot TE, Vasquez AA, Kiemeney LA, Poelmans G (2018). Autism spectrum disorders and autistic traits share genetics and biology. Molecular Psychiatry.

[CR11] Camm-Crosbie L, Bradley L, Shaw R, Baron-Cohen S, Cassidy S (2019). ‘People like me don’t get support’: Autistic adults’ experiences of support and treatment for mental health difficulties, self-injury and suicidality. Autism.

[CR12] Cashin A, Buckley T, Trollor JN, Lennox N (2016). A scoping review of what is known of the physical health of adults with autism spectrum disorder. Journal of Intellectual Disability.

[CR13] Cassidy S, Bradley L, Shaw R, Baron-Cohen S (2018). Risk markers for suicidality in autistic adults. Molecular Autism.

[CR14] Christensen DL, Braun KVN, Baio J, Bilder D, Charles J, Constantino JN, Lee LC (2018). Prevalence and characteristics of autism spectrum disorder among children aged 8 years—autism and developmental disabilities monitoring network, 11 sites, United States, 2012. MMWR Surveillance Summaries.

[CR15] Conner CM, Cramer RD, McGonigle JJ (2019). Examining the diagnostic validity of autism measures among adults in an outpatient clinic sample. Autism in Adulthood.

[CR16] Cooper K, Smith LGE, Russell A (2017). Social identity, self esteem, and mental health in autism. European Journal of Social Psychology.

[CR17] Cooper K, Smith LG, Russell AJ (2018). Gender identity in autism: Sex differences in social affiliation with gender groups. Journal of Autism and Developmental Disorders.

[CR18] Frazier TW, Dawson G, Murray D, Shih A, Sachs JS, Geiger A (2018). Brief Report: A survey of autism research priorities across a diverse community of stakeholders. Journal of Autism and Developmental Disorders.

[CR19] Frith U (2014). Autism—are we any closer to explaining the enigma?. The Psychologist.

[CR20] Gernsbacher MA, Stevenson JL, Dern S (2017). Specificity, contexts, and reference groups matter when assessing autistic traits. PLoS ONE.

[CR21] Hus V, Gotham K, Lord C (2014). Standardizing ADOS domain scores: Separating severity of social affect and restricted and repetitive behaviours. Journal of Autism and Developmental Disorders.

[CR22] Johnson SA, Filliter JH, Murphy RR (2009). Discrepancies between self-and parent-perceptions of autistic traits and empathy in high functioning children and adolescents on the autism spectrum. Journal of Autism and Developmental Disorders.

[CR23] Kapp SK, Gillespie-Lynch K, Sherman LE, Hutman T (2013). Deficit, difference, or both? Autism and neurodiversity. Developmental Psychology.

[CR24] Lord C, Risi S, Lambrecht L, Cook EH, Leventhal BL, DiLavore PC, Rutter M (2000). The Autism Diagnostic Observation Schedule—Generic: A standard measure of social and communication deficits associated with the spectrum of autism. Journal of Autism and Developmental Disorders.

[CR25] Lundström S, Chang Z, Råstam M, Gillberg C, Larsson H, Anckarsäter H, Lichtenstein P (2012). Autism spectrum disorders and autistic-like traits: similar etiology in the extreme end and the normal variation. Archives of General Psychiatry.

[CR26] Lundqvist LO, Lindner H (2017). IS the Autism-Spectrum Quotient a valid measure of traits associated with the autism spectrum? A Rasch validation in adults with and without autism spectrum disorders. Journal of Autism and Developmental Disorders.

[CR27] Massrali A, Brunel H, Hannon E, Wong C, Baron-Cohen S, Warrier V, iPSYCH-MINERvA Epigenetics Group (2019). Integrated genetic and methylomic analyses identify shared biology between autism and autistic traits. Molecular Autism.

[CR28] Milton D, Bracher M (2013). Autistics speak but are they heard?. Medical Sociology Online.

[CR29] Ozsivadjian A, Hibberd C, Hollocks MJ (2014). Brief report: The use of self-report measures in young people with autism spectrum disorder to access symptoms of anxiety, depression and negative thoughts. Journal of Autism and Developmental Disorders.

[CR30] Payne KL, Russell A, Mills R, Maras K, Rai D, Brosnan M (2019). Is there a relationship between cyber-dependent crime, autistic-like traits and autism?. Journal of Autism and Developmental Disorders.

[CR31] Pellicano E, Dinsmore A, Charman T (2014). What should autism research focus upon? Community views and priorities from the United Kingdom. Autism.

[CR32] Pilling S, Baron-Cohen S, Megnin-Viggars O, Lee R, Taylor C (2012). Recognition, referral, diagnosis, and management of adults with autism: Summary of NICE guidance. BMJ.

[CR33] Richards G, Kenny R, Griffiths S, Allison C, Mosse D, Holt R, Baron-Cohen S (2019). Autistic traits in adults who have attempted suicide. Molecular Autism.

[CR34] Ronald A, Hoekstra RA (2011). Autism spectrum disorders and autistic traits: a decade of new twin studies. American Journal of Medical Genetics Part B: Neuropsychiatric Genetics.

[CR35] Ruzich E, Allison C, Smith P, Watson P, Auyeung B, Ring H, Baron-Cohen S (2015). Measuring autistic traits in the general population: A systematic review of the Autism Spectrum Quoteitn (AQ) in a nonclinical population sample of 6,900 typical adult males and females. Molecular Autism.

[CR36] Schriber RA, Robins RW, Solomon M (2014). Personality and self-insight in individuals with autism spectrum disorder. Journal of Personality and Social Psychology.

[CR37] Sizoo BB, Horwitz EE, Teunisse JJ (2015). Predictive validity of self-report questionnaires in the assessment of autism spectrum disorders in adults. Autism.

[CR38] Warner G, Parr JR, Cusack J (2019). Workshop report: Establishing priority research areas to improve the physical health and well-being of autistic adults and older people. Autism in Adulthood.

[CR39] Wilson CE, Roberts G, Gillan N, Ohlsen C, Robertson D, Zinkstok J (2014). The NICE guideline on recognition, referral, diagnosis and management of adults on the autism spectrum. Advances in Mental Health and Intellectual Disabilities.

[CR40] World Health Organization (2018). *International classification of diseases for mortality and morbidity statistics* (11th Revision).

